# Importance of timing optimization for closed-loop applications of vagus nerve stimulation

**DOI:** 10.1186/s42234-023-00110-9

**Published:** 2023-04-27

**Authors:** Ramanamurthy V. Mylavarapu, Vivek V. Kanumuri, Juan Pablo de Rivero Vaccari, Amrit Misra, David W. McMillan, Patrick D. Ganzer

**Affiliations:** 1 Department of Biomedical Engineering, University of Miami, Miami, FL USA; 2 Department of Otolaryngology, University of Miami, Miami, FL USA; 3 The Miami Project to Cure Paralysis, University of Miami, Miami, FL USA; 4 Department of Neurological Surgery, University of Miami, Miami, FL USA; 5 Newton Wellesley Neurology Associates, Newton, MA USA

**Keywords:** Vagus Nerve Stimulation, Closed-loop, Stimulus Timing

## Abstract

In recent decades, vagus nerve stimulation (VNS) therapy has become widely used for clinical applications including epilepsy, depression, and enhancing the effects of rehabilitation. However, several questions remain regarding optimization of this therapy to maximize clinical outcomes. Although stimulation parameters such as pulse width, amplitude, and frequency are well studied, the timing of stimulation delivery both acutely (with respect to disease events) and chronically (over the timeline of a disease’s progression) has generally received less attention. Leveraging such information would provide a framework for the implementation of next generation closed-loop VNS therapies. In this mini-review, we summarize a number of VNS therapies and discuss (1) general timing considerations for these applications and (2) open questions that could lead to further therapy optimization.

## Introduction

Implanted vagus nerve stimulation (VNS) is utilized for a number of applications, including improving cardiovascular function (Ganzer et al. 2022; Kong et al. 2012; Sabbah et al. 2011; Tosato et al. 2006; Tsutsumi et al. 2008; Ugalde, et al. 2014; Vaseghi et al. 2017; Yamaguchi et al. 2018), reducing excessive inflammation (Koopman et al. 2016; Marsal et al. 2021), epilepsy (Elliott et al. 2011a; Elliott et al. 2011b; Alexopoulos et al. 2006; Bauer et al. 2016), and promoting neural plasticity for enhancing motor rehabilitation (Dawson et al. 2021a; Ganzer et al. 2018; Kilgard et al. 2018; Kimberley et al. 2018; Pruitt et al. 2016; Redgrave et al. 2018). Although VNS has been used clinically for more than 30 years, it is not fully understood exactly when VNS should be delivered in relation to disease events (e.g., a seizure) or disease progression (e.g., subclinical or clinical disease) for yielding optimized therapeutic outcomes. Some applications of VNS can use open-loop stimulation (i.e., preprogrammed) for maladies where there is an observable set of symptoms. Increasingly, however, many clinical applications have implemented VNS therapy using closed-loop stimulation (i.e., reactive) “paired” with specific disease events (Tosato et al. 2006; Ganzer and Sharma 2019; Muthiah et al. 2022; Sun and Morrell 2014). In this mini-review, we overview several VNS applications and briefly discuss 2 main considerations for each use of VNS:

- What VNS implementation strategies have worked best (e.g., stimulation timing with respect to disease events or disease progression)?
- Moving forward, what open questions and strategies can be assessed for further optimizing VNS therapy and timing?

## Overview of VNS therapies and timing considerations

**Fig. 1:**
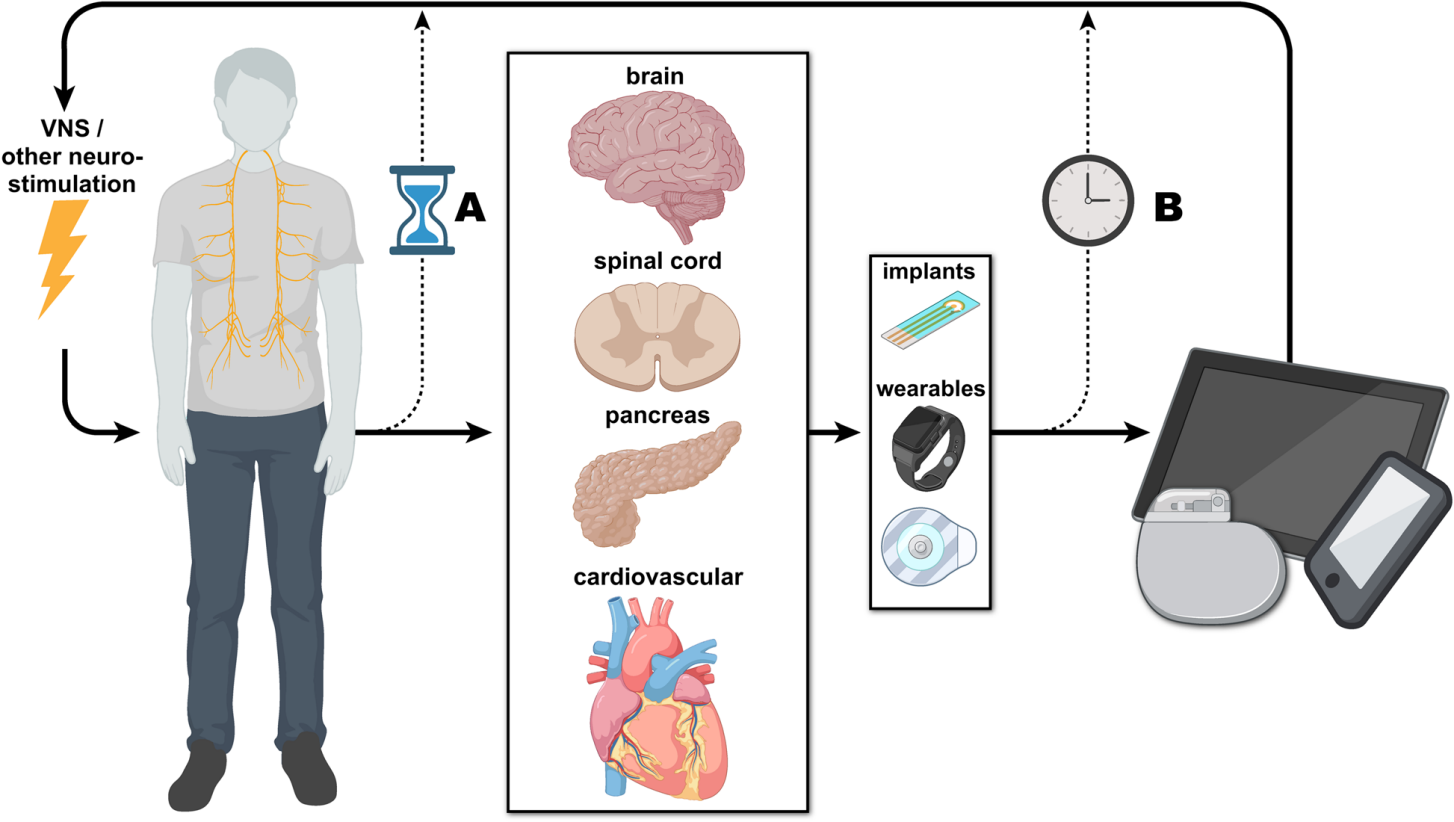
Overview of VNS implementation considerations. This figure illustrates factors that can affect the development of next generation closed-loop VNS therapies. The solid arrows link general VNS implementation considerations. Disease-specific signals can be fed into algorithms on computers or other processing systems to trigger VNS. There are, however, two main considerations involved in determining the optimal timing of VNS (dotted lines): 1) the timing of stimulation with respect to a disease’s progression (**A**, linking the patient and affected systems) and 2) timing of stimulation with respect to recorded disease-specific events (**B**, linking the sensor(s) and stimulation control devices). Multiple paths and design choices can be considered for constructing a VNS system (Created, in part, via Biorender.com)

Below we briefly discuss several well studied applications of VNS therapy and highlight general stimulation timing considerations (Fig. 1). Please see other excellent reviews for a broader summary of VNS applications and general bioelectronic medicines (Ganzer and Sharma 2019; Johnson and Wilson 2018; Pavlov and Tracey 2019; Pavlov and Tracey 2022; Groves and Brown 2005).

## VNS for treating epilepsy

Epilepsy was the first and remains the mostly widely used application for VNS. Initial efforts focused on open-loop stimulation, providing VNS with a duty cycle of 30 s of stimulation followed by 5 min with no stimulation (Labiner and Ahern 2007). Other open loop approaches have seen success treating drug resistant epilepsy with a duty cycle of 30 s on followed by 30 s off administered for just 4 h a day over a chronic period (Bauer et al. 2016).

On the surface, as a disease of recurrent, detectable, paroxysmal events, epilepsy is the archetypical pathology for closed-loop neuromodulation. In the United States, two principal devices are clinically available for closed-loop neuromodulatory treatment of epilepsy: 1) the reactive neurostimulator (RNS) by NeuroPace and 2) VNS with AutoStim Mode by LivaNova. The former uses neural recordings for early seizure detection, while the latter uses relative increases in heart rate (characteristic of seizure activity) for a similar purpose (schematized in Fig. 1B). There remains considerable clinical variability in the response to these timed stimulations, but a subpopulation of epileptic patients demonstrates notable reduction in seizure frequency with these devices. The RNS, following the initial implant cohort over the course of 6 years, showed a 44% reduction in seizure frequency within the first year that increases to 48 – 66% (interquartile range of responders) by 6 years and further to 58–96% by 9 years. Of these, 35% had a greater than 90% reduction in seizure frequency and 73% had a greater than 50% reduction in seizure frequency (Nair et al. 2020; Bergey et al. 2015). Overall, VNS studies have shown that between 59 and 73% of patients newly implanted with closed-loop stimulators show a greater than 50% reduction in seizure frequency (Muthiah et al. 2022; Hamilton et al. 2018).

### Stimulation timing considerations & open questions

Responder rates ranged from 20 and 71% when comparing new responders transitioning from open-loop to closed-loop VNS (Hamilton et al. 2018; Cukiert et al. 2021). Closed-loop neuromodulation is a clinically effective treatment modality. However, it is not clear what biomarkers of pre-ictal and/or ictal events should trigger stimulation. Furthermore, it remains to be determined what the opportunities and challenges are for closed-loop neuromodulation triggered by subclinical seizure events (schematized in Fig. 1A; sustained ictal spiking without any distinct clinical symptoms).

Finally, there is a growing body of evidence suggesting that the true success of closed-loop neuromodulation for epilepsy lies in taking advantage of neural plasticity to re-train cortical networks and give them a lower propensity to seize. In fact, network reorganization, quantified by measurement of frequency dependent functional connectivity, directly correlates with a reduction in seizure frequency (Khambhati et al. 2021). Although promising, it is not yet clear what the closed-loop neuromodulation timing requirements are for promoting beneficial neural plasticity within seizure generating networks.

## VNS & targeted plasticity therapy

Targeted plasticity therapy (TPT) uses brief bursts of closed-loop VNS paired with events to promote neural plasticity and recovery following disease or dysfunction (Hays et al. 2013; Engineer et al. 2019). For example, TPT, using VNS paired with events during rehabilitation, has been used to enhance the effects of sensory or motor therapy following neurological injuries such as stroke, spinal cord injury, and peripheral nerve injury (Dawson et al. 2021a; Kimberley et al. 2018; Redgrave et al. 2018; Darrow et al. 2020a; Darrow et al. 2021; Darrow et al. 2020b; Dawson et al. 2016; Meyers et al. 2018; Meyers et al. 2019; Khodaparast et al. 2016). In a recent pivotal trial, patients with ischemic stroke receiving VNS paired with movements during upper limb rehabilitation showed better clinically meaningful response rates and improved Fugl-Meyer Assessment-Upper Extremity (FMA-UE) scores compared to upper limb rehabilitation alone (Dawson et al. 2021b). These results have now led to Food and Drug Administration (FDA) approval for using TPT (‘VNS + Rehab’) for improving upper limb motor function following ischemic stroke (Commissioner 2021). Although much earlier in clinical testing, similar improvements in upper limb function may be achievable in patients with ischemic stroke receiving noninvasive transcutaneous auricular vagus nerve stimulation (taVNS) paired with movements over an identical timeframe and number of therapy sessions (Redgrave et al. 2018). In addition, TPT has also been used for treating tinnitus, a phantom sensation of sound that is perceived by patients in the absence of any external acoustic stimulus. VNS paired with specific tones (for example those surrounding the frequency of the tinnitus) has shown promise for tinnitus reduction among patients (Vanneste et al. 2017; Tyler et al. 2017). Lastly, a new emerging application of TPT involves pairing VNS with exposure therapy, highly relevant for eventually treating post-traumatic stress disorder (Souza et al. 2022; Noble et al. 2019).

### Timing considerations & open questions

TPT has seen the most success when VNS is precisely paired with specific events (e.g., movements, touch events, auditory tones, and fear extinction; schematized in Fig. 1B). Importantly, benefits are greatly diminished or blocked when VNS is delayed or unpaired (Ganzer et al. 2018; Meyers et al. 2019; Khodaparast et al. 2016). TPT mediated improvements may be most effective when VNS is delivered within a specific time range following the targeted event (Ganzer et al. 2018). This is in agreement with the synaptic eligibility trace theory which states that reinforcement must occur within a given timeframe to effectively modulate neural plasticity (He et al. 2015). Lastly, several applications of TPT currently involve outpatient therapy sessions in a clinical setting. What additional benefit can be achieved, if any, when TPT is delivered outside of the clinic during daily activities and how can this be achieved across different applications of TPT?

## VNS for treating type 2 diabetes

Type 2 diabetes is one of the most common causes of morbidity in the world and leads to the development of peripheral neuropathies, vasculopathy, and other sequalae over time (Khan et al. 2020; Zhang et al. 2020). VNS has been explored for treatment of both diabetes itself and potentially its broader complications (Payne et al. 2020; Huang et al. 2014). In particular, VNS has been used to treat diabetes-related neuropathies for pain, and is in clinical trials to address some symptoms of diabetes with complications from autonomic neuropathy (Li et al. 2018; Okdahl et al. 2021). Finally, VNS with short-pulse width at 5 Hz has been used to decrease blood glucose and improve glucose tolerance in animal models (Yin et al. 2019). For example, in a minipig model, VNS did not significantly modulate metabolic rates while attenuating both gain of total-body and fat mass (Sobocki et al. 2006). Indeed, bilateral VNS over 12 weeks showed improvements in glucose uptake and insulin sensitivity in comparison to minipigs not receiving VNS (Malbert et al. 2017). This information provides a robust basis for the provision of VNS therapy over chronic periods in treatment of type 2 diabetes.

### Stimulation timing considerations & open questions

The artificial pancreas is one of the most cutting-edge closed-loop technologies for treating diabetes. It uses continuous glucose monitoring, an algorithm, and an insulin pump for affecting glucose levels (Boughton and Hovorka 2019). Closed-loop VNS and other neuromodulation approaches could also see success in targeting metabolic events during diabetes (Güemes Gonzalez et al. 2020). However, it is not clear what exact metabolic processes – those enacted via neural, endocrine, or mixed vectors—should be targeted with closed-loop neuromodulation and whether this would provide further benefit (schematized in Fig. 1B). For example, there is evidence that intermittent VNS can provide more potent effects compared to continuous stimulation (Yin et al. 2019), highlighting that VNS timing may be important for treating type 2 diabetes.

A particular timing challenge with type 2 diabetes is that it is a chronic, progressive clinical condition. It is unclear exactly when in the progression of this disease VNS might be most effective (schematized in Fig. 1A). There is also a significant period where this malady is subclinical, as the spikes in blood glucose and A1C levels do not meet the requirements for clinical intervention. The possibility of using VNS in these periods, and the effects of approaching such subclinical use cases, is an intriguing avenue of future research to potentially help prevent the progression type 2 diabetes. Recent efforts have begun focusing on potentially treating subclinical diabetes / prediabetes (Braga et al. 2019).

## VNS for controlling inflammation

VNS has mainly been used to treat inflammatory conditions, such as rheumatoid arthritis, through open-loop stimulation protocols (Koopman et al. 2016; Marsal et al. 2021). Please see the following excellent reviews for details on the role of VNS in regulating inflammation (Bonaz et al. 2013; Bonaz et al. 2016; Czura et al. 2003; Pavlov and Tracey 2012; U., et al. 2022; Hilderman and Bruchfeld 2020; Falvey et al. 2021). VNS regulates the inflammatory response through the cholinergic anti-inflammatory pathway (CAP (Bonaz et al. 2013; Bonaz et al. 2016; Czura et al. 2003)). For example, stimulation of the efferent vagus nerve can mediate acetylcholine release which then interacts with macrophages to inhibit the production of pro-inflammatory cytokines such as interleukin (IL)-1, IL-6 and tumor necrosis factor (TNF) (Bonaz et al. 2016; Borovikova et al. 2000; Ulloa 2005; Das 2007). Importantly, with therapeutic VNS, different combinations of pulse width, pulse amplitude, and frequency produce different effects on cytokine levels. For example, *Tsaava *et al*.* showed that stimulation at the pulse width of 50 μs at 30 Hz and 200 μA, as well as stimulation with a 50 μs pulse width at 100 Hz and 750 μA, lowered the levels of TNF in serum of normal mice. However, increasing the pulse width at the lower frequency with higher amplitude of stimulus had the opposite effect (Tsaava et al. 2020). Similarly, stimuli with low pulse widths at 30 Hz increased the levels of IL-10 (an anti-inflammatory cytokine) in serum regardless of amplitude (Tsaava et al. 2020). Taken together, these findings indicate that select VNS parameters can differentially affect the inflammatory milieu. Similarly, VNS decreases infiltration of immune cells such as neutrophils into sites of damage. The effects on neutrophils are likely due to the short-term duration of some of the VNS protocols used experimentally, considering that neutrophils are the first immune cells to usually migrate to a site of injury or disease. Therefore, it is possible that longer duration protocols similarly affect other immune cells.

### Stimulation timing considerations & open questions

Although open-loop VNS has seen success in mitigating inflammation, it is not clear under what conditions closed-loop VNS could provide further benefit. The dynamics of inflammatory processes are complex and will require extensive further study to identify appropriate biomarkers to guide optimal timing of stimulation (schematized in Fig. 1B). Several recent efforts have begun using neurogram decoding (Zanos 2019) and biosensing (Lu et al. 2021; Rothbauer et al. 2020; Kanazawa et al. 2016) to detect changes in inflammatory biomarkers. In addition, it has been suggested that VNS therapy may play a beneficial role in depression, which has an inflammatory component (Syed et al. 2018) by inhibiting the production of pro-inflammatory cytokines (Das 2007). Similarly, in a model of continued stress, VNS (10 mA, 5 Hz, 5 ms of pulse duration for 5 min) decreases the levels of caspase-3, TNF, IL-1β and IL-6 in the hippocampus (Namgung et al. 2022). The specific biomarkers and events, if any, that can be targeted for mitigating the inflammatory contributions to depression and/or stress has not been reported on extensively.

## VNS for treating cardiovascular conditions

Cardiovascular disease is the leading cause of morbidity and mortality worldwide (World Health Organisation, 2018). VNS is a potentially useful therapy for cardiovascular disease due to a number of factors, including the presence of inflammation, enhanced sympathetic tone, and a direct effect of VNS on cardiovascular tissues (Capilupi et al. 2020; Ottaviani et al. 2022). VNS is now being used to treat experimental or clinical cardiovascular disease and associated conditions (Yamaguchi et al. 2018; Capilupi et al. 2020; Ottaviani et al. 2022; DiCarlo et al. 2018; Premchand et al. 2014; Anand et al. 2020). The majority of VNS to date involves open-loop protocols for cardiovascular therapy. Open-loop VNS may confer therapeutic benefit through several mechanisms, including enhancing myocardial electrical stability, modulating chronotropy, decreasing inflammation, and increasing parasympathetic tone (Capilupi et al. 2020; Ottaviani et al. 2022).

There are now several studies investigating the potential of closed-loop VNS for treating cardiovascular conditions (Ganzer et al. 2022; Tosato et al. 2006; Ottaviani et al. 2022; Ferrari et al. 2011). For instance, closed-loop stimulation in a porcine model used the RR interval to power VNS parameters, with physiological changes being maintained up to a few minutes (Tosato et al. 2006). In humans, heart related signals were similarly used to deliver VNS with a variable delay of up to 325 ms from the R-wave of the electrocardiogram (Ferrari et al. 2011). This timing of stimulation resulted in significant improvements in patient quality-of-life measures which continued up to 1 year (Ferrari et al. 2011). Such information brings to light the importance of comparing open-loop and closed-loop VNS delivery. Furthermore, use of RR interval, along with several other cardiovascular biomarkers, are potential sources for triggering closed-loop VNS. Please see other excellent reviews for a broader assessment of VNS therapy for cardiovascular conditions (Capilupi et al. 2020; Ottaviani et al. 2022).

Closed-loop VNS could potentially treat spontaneous myocardial ischemia, via dynamic detection of ischemic events triggering stimulation. Myocardial ischemia is involved in several cardiovascular conditions, where there is a prolonged imbalance of myocardial oxygen supply and demand (Buja 2005). One recent study has demonstrated the feasibility of providing closed-loop VNS during myocardial ischemia events triggered by a machine learning model (Ganzer et al. 2022). In a subset of experiments, myocardial ischemia was induced by a high dose infusion of catecholamines leading to correlates of myocardial ischemia, including ST segment depression and arrythmias. Closed-loop VNS was applied for the remainder of the induced ischemic state once triggered by the machine learning model (Ganzer et al. 2022). Closed-loop VNS significantly mitigated several correlates of myocardial ischemia, whereas open-loop VNS had modest to no beneficial effect (Ganzer et al. 2022). This pairing of machine learning and recorded signals is an example of how timing can be utilized to improve VNS delivery (schematized in Fig. 1B).

### Stimulation timing considerations & open questions

There are a number of spontaneous events that can potentially trigger closed-loop VNS for therapeutic benefit in the setting of cardiovascular disease. Regardless, it is not readily apparent that a reactive and closed-loop treatment is necessarily optimal for treating spontaneous cardiovascular disease events. Pharmacological medicines, stents, coronary bypass grafts, ablation for atrial fibrillation, and other techniques can confer significant therapeutic benefit. The factors that contribute to cardiovascular disease are exceedingly complex and may not involve rapid spontaneous events relevant for closed-loop VNS (e.g., chronic inflammation or poor lifestyle choices). Overall, the ultimate role of closed-loop VNS for treating cardiovascular disease will be affected by a number of exciting innovations, including neurogram decoding of cardiovascular information (Zanos 2019; Ottaviani et al. 2022) and machine learning triggered VNS using wearable or implanted cardiovascular sensors (Ganzer et al. 2022; Sun et al. 2022). Modern pacemakers now incorporate closed-loop features to enable reactive pacing based on physiological state (Świerżyńska, et al. 2023). The therapeutic efficacy of closed-loop cardiac rhythm management devices will be an exciting area to also watch, regarding the effective design and promise of closed-loop therapies for cardiovascular dysfunction.

## Potentially using VNS for treating subclinical disease

While therapeutic intervention for disease states that are symptomatic (i.e., clinical) but not perceivable is important, such disease progression is usually preceded by a series of interconnected subclinical states (asymptomatic or not meeting the threshold for clinical intervention; schematized in Fig. 1A). There is substantial evidence that the subclinical states underlying future medical conditions are detectable. Chronic subclinical inflammation, for example, has been linked as a risk factor in the development of diabetic polyneuropathies (Herder et al. 2009). Additionally, subclinical hypercortisolism and hypothyroidism have been linked to increased incidence of type 2 diabetes and gestational diabetes, respectively (Chiodini et al. 2005; Tudela et al. 2012). Lastly, plasma concentrations of inflammatory markers have shown a correlation with risk of future stroke and myocardial infarctions (Ridker et al. 1997). Future research will need to identify the opportunities and challenges associated with treating subclinical disease, especially in the absence of specific medical treatment guidelines.

## Conclusions

VNS therapy has been effective across many clinical applications; however, there remains a need for detailed analyses regarding the optimal timing of this treatment to maximize clinical benefits. Current VNS delivery with respect to overall disease progression and specific disease related events can vary widely across applications. The advent of novel biosensing and machine learning provides a method to potentially (1) detect disease events and better time VNS and (2) intervene earlier in disease progression to maximize the clinical benefits of VNS (Fig. 1). These key points will be imperative to maximizing the efficacy of next generation closed-loop VNS therapies.

## Data Availability

N/A.

## Abbreviations

VNS: Vagus Nerve Stimulation
TPT: Targeted Plasticity Therapy
FMA-UE: Fugl-Meyer Assessment – Upper Extremity
FDA: Food and Drug Administration
taVNS: Transcutaneous Auricular Vagus Nerve Stimulation
RNS: Reactive Neurostimulator
CAP: Cholinergic Anti-Inflammatory Pathway
IL: Interleukin
TNF: Tumor Necrosis Factor
ECG: Electrocardiogram
